# A case report of lymphangioleiomyomatosis with retroperitoneal masses in pregnancy

**DOI:** 10.3389/fmed.2023.1313503

**Published:** 2023-12-22

**Authors:** Yashi Zhu, Chao Wang, Jianyi Ding, Meiqin Yang, Yin Bo, Mingjun Ma, Haoran Hu, Jiejun Cheng, Lingfei Han, Yu Wang

**Affiliations:** ^1^Department of Gynecology, Shanghai First Maternity and Infant Hospital, School of Medicine, Tongji University, Shanghai, China; ^2^Department of Radiology, Shanghai First Maternity and Infant Hospital, School of Medicine, Tongji University, Shanghai, China

**Keywords:** retroperitoneal lymphangioleiomyomatosis, pregnancy, preoperative diagnosis, differential diagnosis, case report

## Abstract

**Background:**

Lymphangioleiomyomatosis (LAM) is a rare, gradually advancing tumor of unknown origin. It is distinguished by the anomalous proliferation of pulmonary smooth muscle cells and predominantly manifests in women of childbearing age. In this study, we aim to present a noteworthy case of LAM accompanied by lymphangioleiomyoma in the retroperitoneal space during pregnancy, a scenario susceptible to misdiagnosis.

**Case presentation:**

A 31-year-old woman, facing an unintended pregnancy, presented during the 13th week with a cystic-solid mass exhibiting abundant blood signals in the pelvic cavity, as revealed by routine obstetrical ultrasound. Concurrently, her chest CT disclosed diffuse thin-walled cavities in both lungs. Despite the absence of clinical symptoms, the patient abandoned pregnancy and underwent a complete curettage. However, 24 days post-operation, she was readmitted for further assessment, revealing an enlargement of the mass encompassing the abdominal aorta and inferior vena cava, along with compression on the middle and lower segments of the ureter. After a multi-disciplinary discussion and patient explanation, an exploratory laparotomy was performed, resulting in the complete removal of the tumor. Intraoperative pathological examination and immunohistochemical staining indicated a retroperitoneal mass devoid of malignant evidence. The comprehensive morphologic and immunophenotypic features substantiated the diagnosis of lymphangioleiomyomatosis. The postoperative course was uneventful, culminating in the patient’s discharge.

**Conclusion:**

The consideration of Lymphangioleiomyomatosis (LAM) with a retroperitoneal tumor is crucial in the differential diagnosis of pelvic and abdominal masses. The preoperative diagnosis of this tumor poses a challenge, as ultrasound or CT scans may not yield definitive results. Accurate diagnosis necessitates not only a pathological examination of the retroperitoneal mass but also the correlation with the patient’s chest High-Resolution Computed Tomography (HRCT) findings and corresponding clinical manifestations. Optimal management involves radical surgery, with surgeons comprehensively factoring in both fetal and maternal conditions when formulating a treatment plan.

## Background

Lymphangioleiomyomatosis (LAM) is a rare, slowly progressive tumor of unknown etiology, almost only occurring in women of childbearing age (1). Hence, consideration of the patient’s pregnancy becomes imperative. The prevalence of LAM among women has been estimated at 5 per 1,000,000 (2). Microscopically, LAM is distinguished by abnormal hyperplasia of smooth muscle cells in the lungs, lymphatic vessels, and mediastinal, abdominal, and lower cervical lymph nodes (3). The primary site of occurrence is the lungs, most patients experience pulmonary symptoms, including pneumothorax, chest pain, cough, and pleural effusion (4). Despite the rarity of extrapulmonary LAM, instances have been reported in the mediastinum and retroperitoneum, often accompanied by pulmonary invasion.

The retroperitoneal space, also referred to as the retroperitoneal cavity, is situated in the posterior abdomen. It serves as the encompassing term for the space and internal anatomical structure positioned between the parietal peritoneum and the transverse fascia of the abdomen, extending from the septum to the pelvic septum (5). Organs situated within this space include the pancreas, a segment of the duodenum, adrenal glands, kidneys, ureters, and more. The retroperitoneal space also accommodates the abdominal aorta, inferior vena cava, and their tributaries, along with nerve trunks (sympathetic and spinal nerves), lymph nodes, lymphatic vessels, and a substantial amount of loose connective tissue, fat, muscle, fascia, embryonic residual tissue, and remnants of the primitive urogenital ridge. These diverse elements may serve as potential sources of tumors. Owing to the prevalence of loose connective tissue in this space, tumors can remain asymptomatic for an extended duration until they attain significant volume and exert compression on surrounding nerves, blood vessels, gastrointestinal, and urinary organs. Notably, when presenting as palpable abdominal or pelvic masses, these tumors can be mistaken for conditions such as ovarian cancer, adnexal masses, renal cysts, and adrenal tumors. Without due consideration of the possibility of this disease, clinicians may encounter challenges in diagnosis.

Given its rarity and challenging preoperative diagnosis, we report an unusual case involving a 31-year-old pregnant female diagnosed with lymphangioleiomyomatosis.

## Case report

A 31-year-old woman with unintended pregnancy was diagnosed during a routine obstetric ultrasound performed in the 13th week of gestation with a mixed mass located in the bilateral pelvic cavity. The patient had regular menstrual cycles, and diligently employed contraceptive measures, such as using condoms, during sexual activities. In terms of pregnancy history, the patient had experienced only one full-term delivery, without any instances of premature birth or miscarriage. Notably, in the previous delivery, the patient opted for a cesarean section due to the substantial size of the baby.

**Figure 1:**
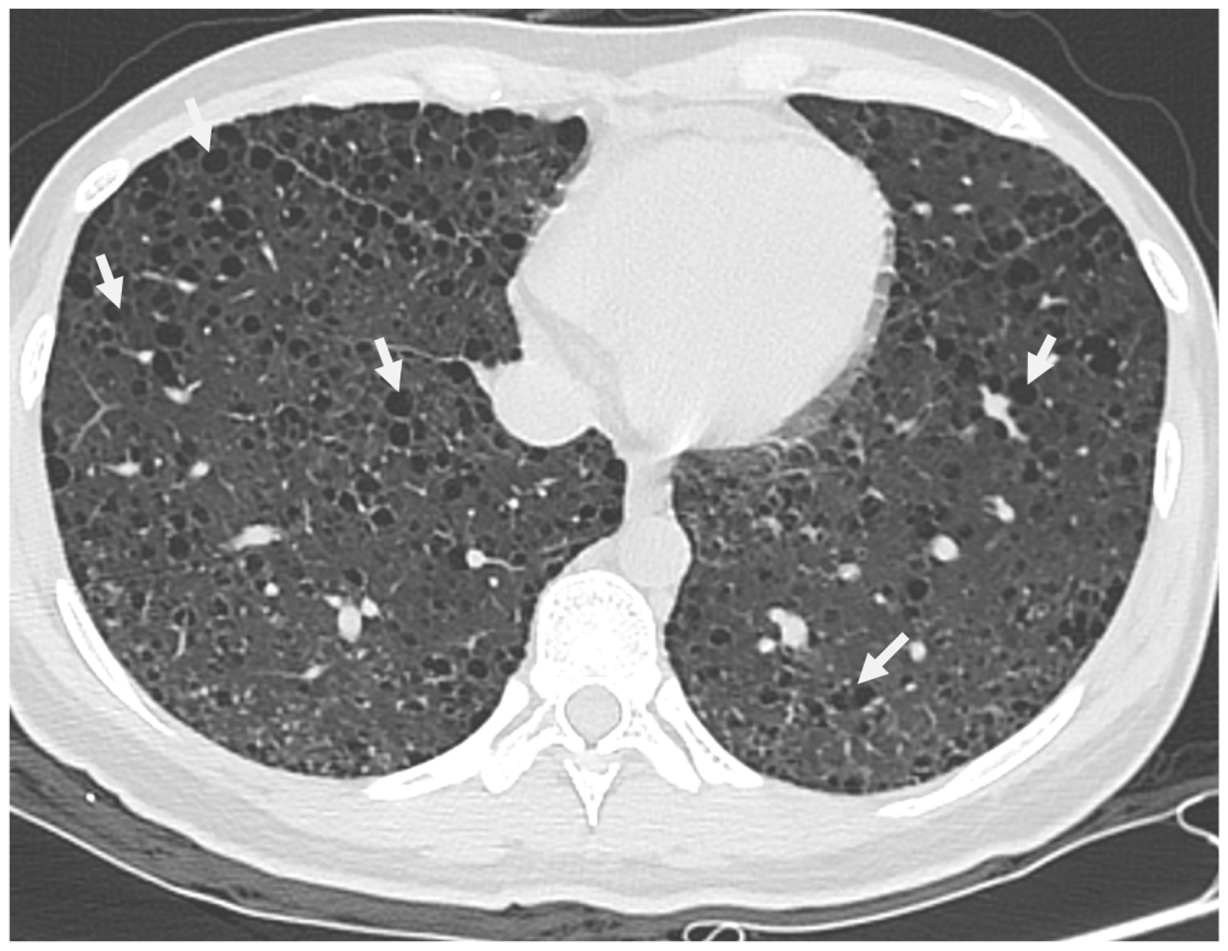
Chest CT scan in a pregnant woman seen at Shanghai First Maternity and Infant Hospital Affiliated with Tongji University. The white arrow points to the thin-walled cystic changes in both lungs.

Upon physical examination, her abdomen was soft, but a mass was felt in her left adnexal area with no tenderness. Upon ultrasonography, the uterus was normal in size with a well-defined edge and the myometrium was heterogeneous. Moreover, the left adnexa, the left, and right iliac fossa, and the right-posterior uterus all showed cystic masses with solid lesions of 46*44*39, 30*72*73, 41*59*60, and 43*87*93 mm, respectively. Color doppler flow imaging (CDFI) revealed rich blood flow signals in the above areas. It was also found that the left and right omentum thickened by 17 and 11 mm, respectively. According to these, the sonographer suspected that the patient had mixed pelvic masses, uterine myomas, and adnexal masses. Her chest CT showed diffuse thin-walled cystic changes in both lungs (Figure 1). What is worth mentioning is the laboratory analyses showed an elevated Alpha-fetoprotein level (17.19 ng/mL). Moreover, the patient’s serum level of cancer antigen 125 (CA-125) was elevated at 50.70 U/mL. Her serum levels of cancer antigen 19–9 (CA19-9) and cancer antigen 153 (CA-153) were 12.95 and 23.70 U/mL, respectively, and both were within normal values. Her serum level of premenopausal and postmenopausal ROMA was 7.61 and 23.67%, which were also normal.

Following admission, the patient sought termination of pregnancy and underwent comprehensive pre-surgical examinations. Subsequently, she underwent curettage under B-ultrasound monitoring. The procedure transpired without complications, and the postoperative course was uneventful, leading to the patient’s subsequent discharge.

To promptly ascertain the nature and origin of the pelvic mass, the patient underwent positron emission tomography/computed tomography (PET-CT) on the 12th day postoperatively. The examination results revealed the following: a cystic-solid shadow of soft tissue in the left adnexal area and multiple cystic shadows with low density appearing in the retroperitoneum and on both sides of the pelvic cavity. Enlargement of the left inguinal lymph nodes was noted, accompanied by increased fluorodeoxyglucose activity, whereas the right inguinal lymph nodes, intestines, and stomach exhibited normal fluorodeoxyglucose activity. These findings suggested a probable malignancy originating from the left adnexa and its metastasis.

24 days after the curettage, the patient was admitted to our hospital again. During the ultrasonography examination, a marked increase in the mixed echo of the left adnexa was observed compared to the previous examination, measuring 81*84*78 mm. Similarly, an increased mixed echo was detected in the right-posterior uterus, measuring 43*109*106 mm. The mixed echo in left iliac fossa measured 20*60*64, smaller than before, while the echo of right iliac fossa remained unchanged. Rich intralesional vascularization was evident in these areas.

**Figure 2:**
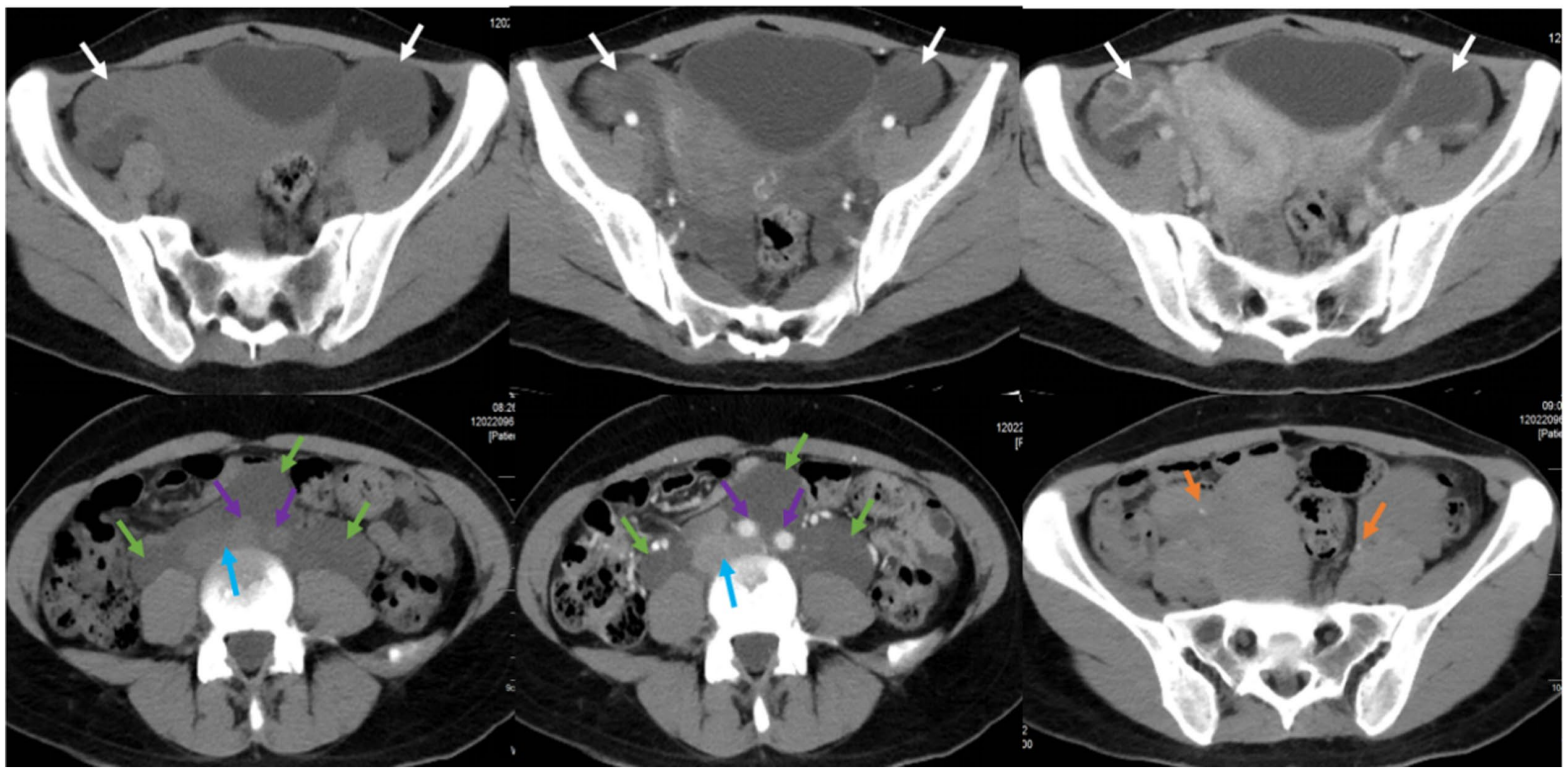
Pelvic CT plain scan and enhancement in a pregnant woman seen at Shanghai First Maternity and Infant Hospital Affiliated with Tongji University. The white arrow points to bilateral ovaries, the green arrow points to the cystic-solid mass, the blue arrow points to the abdominal aorta, the purple arrow points to the inferior vena cava and iliac vessels, and the yellow arrow points to the ureters.

**Figure 3:**
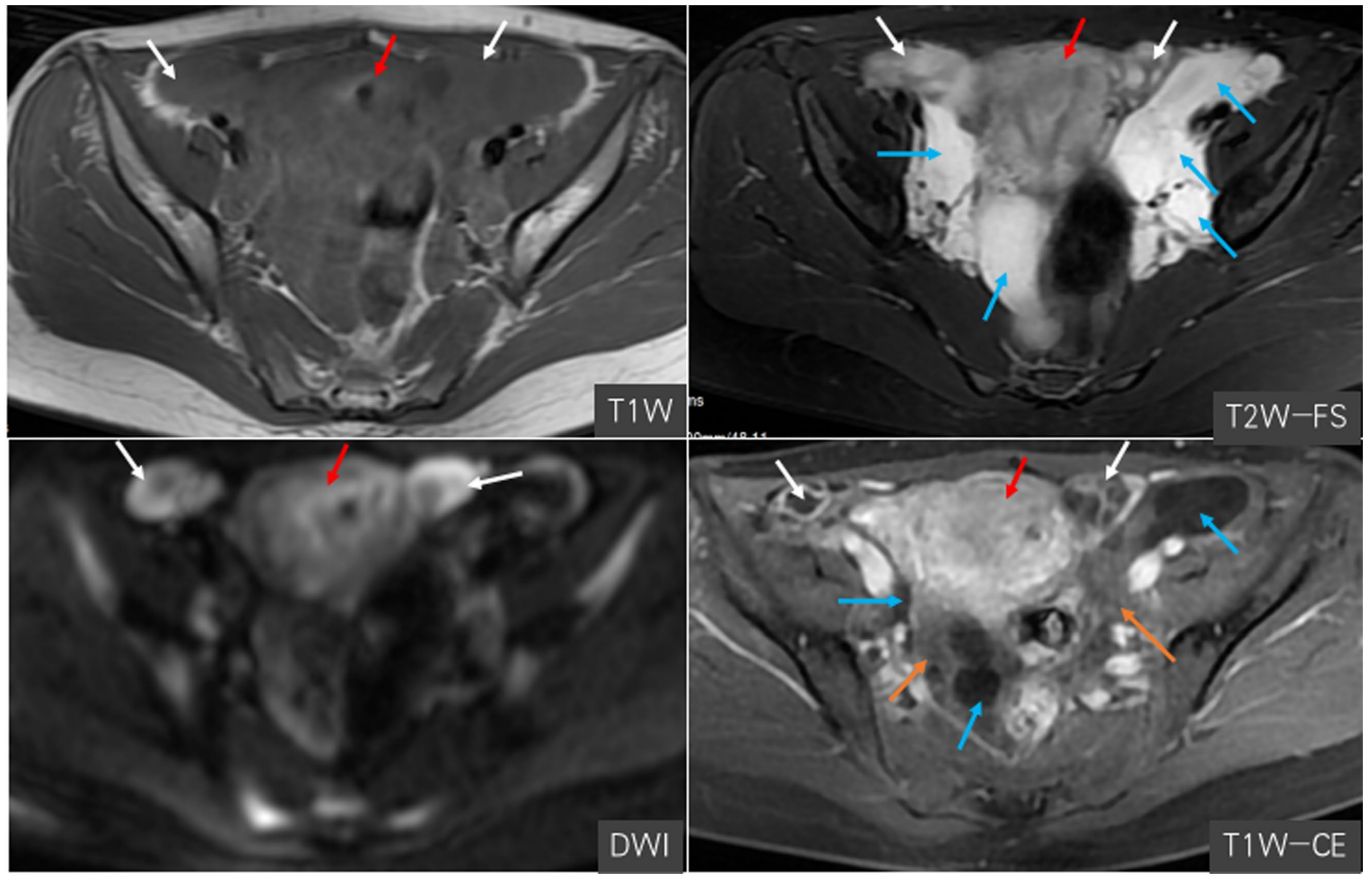
Magnetic resonance imaging in a pregnant woman seen at Shanghai First Maternity and Infant Hospital Affiliated with Tongji University. The white arrow points to bilateral ovaries, the blue arrow points to the cystic-solid mass, the red arrow points to the uterus, and the yellow arrow points to the vessels.

**Figure 4:**
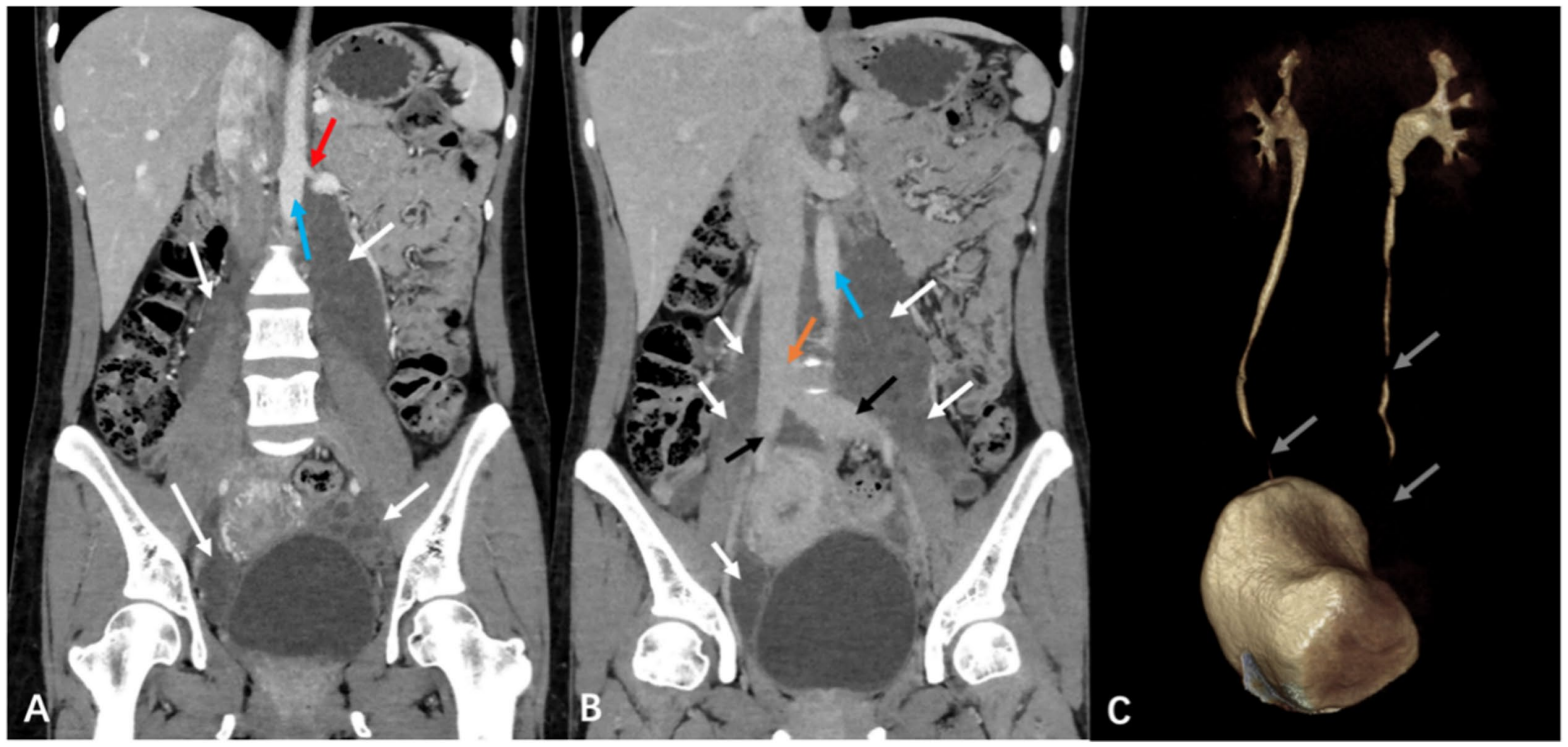
Computed tomography urography (CTU) and three-dimensional reconstruction in a pregnant woman seen at Shanghai First Maternity and Infant Hospital Affiliated with Tongji University. The white arrow points to the mass, the red arrow shows its upper, the blue arrow points to the abdominal aorta, the yellow arrow points to the inferior vena cava, and the gray arrow points to the middle and lower part of the ureters.

Furthermore, the patient underwent additional imaging evaluations, including pelvic enhanced CT (Figure 2), enhanced pelvic Magnetic Resonance Imaging (MRI; Figure 3), computed tomography urography (CTU), and three-dimensional reconstruction (Figure 4). The results revealed heterogeneous enhancement of bilateral ovaries and a widespread cystic-solid mass in the pelvic and retroperitoneum. The upper edge of the cystic part reached the level of the renal artery, wrapping around the abdominal aorta, inferior vena cava, iliac artery, and vein, while embedding the middle and lower part of the ureter. Importantly, the shape and density of both kidneys were normal.

A specialized medical committee, comprising an oncological gynecologist, surgeon, anesthetist, radiologist, and pathologist, convened to deliberate on the optimal therapeutic strategy for this unique case. The ultrasound examination results indicated a pronounced and highly active proliferation of the tumor. Magnetic resonance imaging and CT examinations revealed the tumor’s encasement around major blood vessels in the pelvic and abdominal cavities, suggesting a potential risk of vascular compression and rupture, leading to severe bleeding. Considering the aggressive nature of the tumor, conservative treatments such as medication were deemed likely to cause delays in the condition. Consequently, the committee recommended exploratory laparotomy and thorough radical resection based on intraoperative pathological examination as the most effective treatment approach.

**Figure 5:**
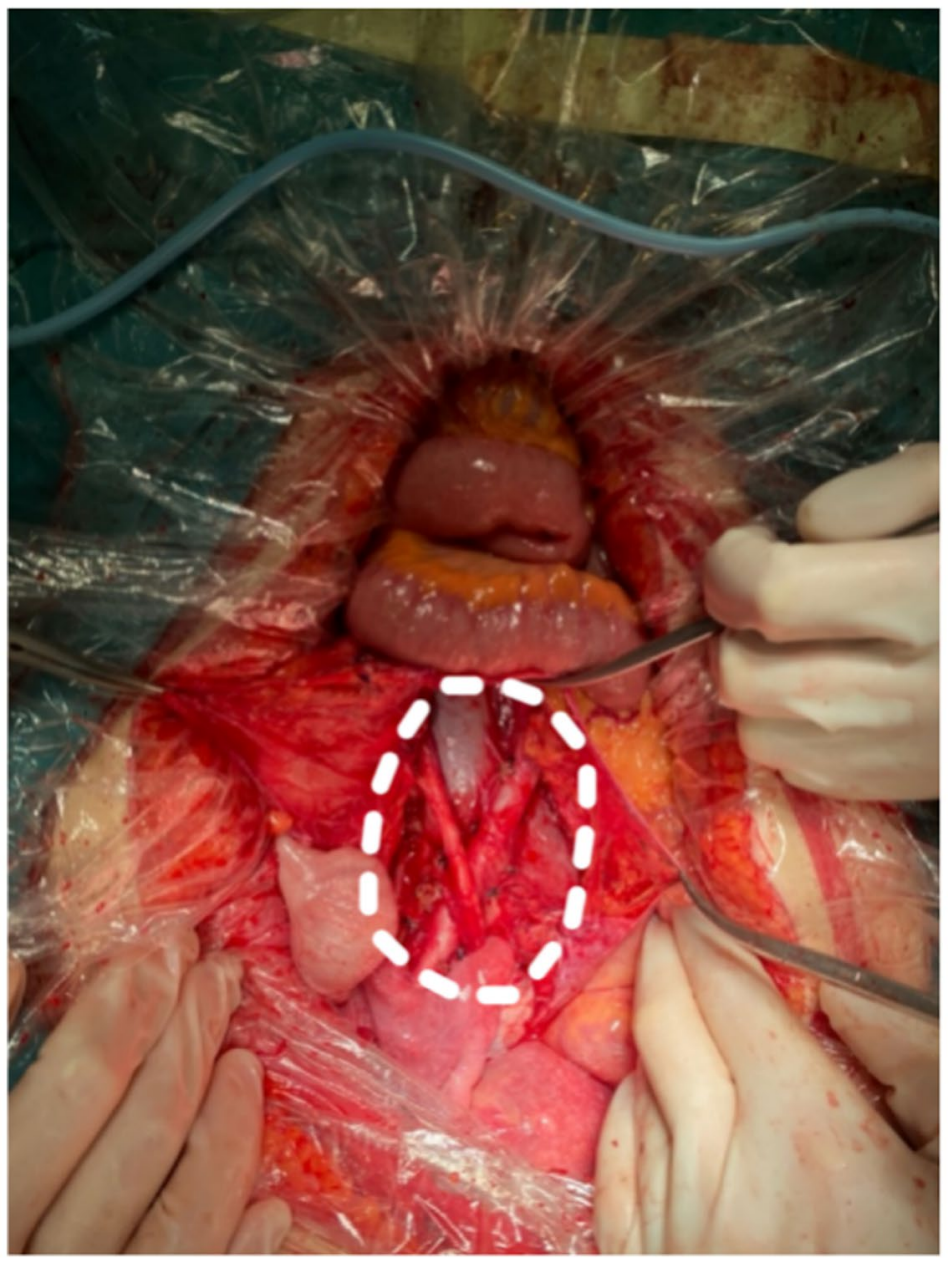
Complete resection of retroperitoneal tumor. The white dashed line circles the original tumor location.

Subsequently, the patient underwent bilateral ureteral catheterization under a cystoscope. Intraoperatively, in alignment with imaging evaluations, masses were identified in the retroperitoneum, near the abdominal aorta, bilateral iliac vessels, and the left inguinal canal, encompassing bilateral ureters. The lesions measured 155 and 107 cm in size, with a yellow cystic mass of 433 cm observed in the posterior wall of the uterus near the cervix, tightly adhering to the cervix. Importantly, no other pathological aspects were noted during the laparoscopic exploration of the abdominal and pelvic cavities. The intraoperative pathological examination confirmed that the retroperitoneal mass consisted of spindle cells and numerous irregular lymphatic vessels resembling lacunae, with no evidence of malignancy. Consequently, resection of retroperitoneal and cervical lesions was performed (Figure 5).

Immunohistochemical staining results, as presented in Table 1, demonstrated the positivity of neoplastic cells for Vimentin, Smooth-Muscle-Actin, Desmin, and Caldesmon, indicating a likely origin from mesenchymal tissue. Subsequent analysis revealed HMB-45 (partially positive) and Melan-A (negative), effectively excluding the possibility of malignant melanoma. Furthermore, negative results for S100, CK pan, and EMA suggested non-neural and non-epithelial tissue origin. Notably, positive staining for D2-40 (vessel +), CD34 (+), and CD31 (+) indicated lymphatic infiltration and the potential for differentiation into vascular endothelium. Ki-67, a marker of cell proliferation, exhibited a positive value of 5%, suggesting a higher likelihood of benign tumors with a lower proliferation rate and a more favorable prognosis. Importantly, the results for estrogen receptor (ER) and progesterone receptor (PR) were positive. In summary, the comprehensive analysis of morphologic and immunophenotypic features strongly supported the diagnosis of lymphangioleiomyomatosis.

**Table 1:** The result of immunohistochemical staining.

Immunohistochemical marker	Clinical significance
Viementin (+), SMA (+), Desmin (+), and Valdesmon (+)	Source of mesenchymal tissue
HMB (part+), Melan-A (−)	Unlikely to be melanoma
S100 (−)	Unlikely to originate from neural tissue
CK pan (−), EMA (−)	Unlikely to originate from epithelial tissue
Ki-67 (+5%)	Inactive proliferation
D2-40 (vessel+), CD34 (+), and CD31 (+)	Vascular invasion and differentiation
ER (part+), PR (+)	Indicate a good prognosis

The postoperative course proceeded without complications, and the patient was discharged on the eleventh day following the procedure. We recommend that the patient attend the LAM clinic for further treatment. During the 17-month follow-up, there was no evidence of recurrence or disease progression.

## Discussion and conclusion

Lymphangioleiomyomatosis (LAM) is a rare tumor disease primarily afflicting premenopausal women, with an average age of diagnosis at 41 years (6). It is characterized by cystic lung destruction, abdominal tumors, and the accumulation of chylous fluid due to infiltration of neoplastic LAM cells (7). The prevalence of LAM is estimated to affect approximately 3.4–7.8 per million women (2). Its pathological hallmark is abnormal smooth muscle cell hyperplasia in the lung, along with involvement in the lymphatic systems of the mediastinum and retroperitoneum (3). The diagnosis of this disease can be aided by CT findings, revealing multiple thin-walled cysts uniformly distributed throughout the lung (8). A common clinical presentation of LAM is spontaneous pneumothorax in women aged 40–50 years. Consequently, LAM is frequently misdiagnosed as asthma or chronic obstructive disease, leading to delayed diagnosis (7).

Extrapulmonary lymphangioleiomyomatosis (eLAM) is a rare occurrence, with 16% of cases localized in the retroperitoneum (9). Retroperitoneal LAM may exhibit clinical features ranging from asymptomatic cases to manifestations such as abdominal distension, edema, urinary system symptoms, or acute abdomen (10). In our case, the patient was LAM with retroperitoneal lymphangioleiomyoma, displaying a palpable abdominal mass and enlargement of inguinal lymph nodes. Chest CT revealed diffuse thin-walled cystic cavities in both lungs, aligning with characteristic lung imaging changes associated with LAM. However, due to a limited understanding of this disease, our focus was primarily on the tumor itself preoperatively, leading to an oversight of chest CT findings. Despite the absence of pulmonary symptoms, potentially due to compensatory mechanisms within the body, regular physical examinations and follow-ups remained essential.

The occurrence of retroperitoneal Lymphangioleiomyomatosis (LAM) during pregnancy is exceptionally rare, posing a significant challenge for obstetricians, gynecologists, radiologists, and surgeons in terms of preoperative diagnosis. Firstly, when presenting as an abdominal or pelvic mass, retroperitoneal LAM is highly prone to confusion with metastatic tumors, ovarian cancer, adnexal cysts, or pancreatic cysts. Secondly, the positioning of retroperitoneal LAM between vascular structures can lead to misdiagnosis as lymphangioma through CT or ultrasound examinations (11). Even from a histological perspective, distinguishing it from other lymphatic diseases is challenging (12). The presence of lymph node enlargement also complicates the exclusion of malignant pelvic tumors. Lastly, as part of the PEcoma group (13), there exists morphological overlap between LAM and uterine PEcoma. Both conditions may affect individuals in the same age group and demonstrate an association with tuberous sclerosis complex (TSC) (14). However, PEcomas are immunoreactive for melanocytic and smooth muscle markers, displaying both HMA45(+) and Melan(+) (15–17).

As a specialized hospital focusing on gynecological tumors, our initial assumption regarding the retroperitoneal mass leaned toward a gynecological disease. In this specific case, the patient was initially suspected of having an ovarian tumor until postoperative pathological and histological examination results were obtained. Upon retrospective analysis and literature review, it became evident that Lymphangioleiomyomatosis (LAM) cells exhibit characteristics of smooth muscle cells, expressing smooth cell actin, vimentin, and desmin. Moreover, they display features not present in normal muscle cells, such as the expression of melanoma-related proteins (HMB-45) and receptors for estrogen and progesterone (18). Numerous clinical studies have highlighted vascular endothelial growth factor D (VEGF-D) as a biomarker for the progression and treatment response of LAM (19–21). In specific clinical conditions, detecting VEGF-D may obviate the need for lung biopsy in LAM patients (19). Since we did not consider the possibility of LAM before surgery, we did not test the patients’ serum VEGFD concentration. More importantly, according to the diagnostic criteria for Lymphangioleiomyomatosis published by guidelines (22), high-resolution computed tomography (HRCT) plays an irreplaceable role in diagnosing LAM. When characteristic imaging emerges, due consideration should be given to the possibility of LAM. However, in asymptomatic LAM patients, relying solely on HRCT imaging findings may be insufficient; the diagnosis necessitates the involvement of experienced pathologists and appropriate immunohistochemical markers.

For patients with retroperitoneal tumors where the possibility of malignancy cannot be ruled out, a recommended course of action is a radical surgical resection performed by experienced surgeons (23). In our specific case, the retroperitoneal tumor exhibited an increasing trend, making it challenging to determine its nature. Opting for medical or conservative treatment might risk missing the optimal window for the best treatment opportunity.

In recent years, substantial progress has been achieved in our understanding and management of Lymphangioleiomyomatosis (LAM) through the development of clinical trials and basic research, significantly enhancing patient prognosis. Beyond surgical interventions, pharmacological treatments have also demonstrated effectiveness. The mTORC1 inhibitor sirolimus (rapamycin) has emerged as a potential treatment, given the pivotal role of the mTORC1 signaling pathway in the pathogenesis of LAM (24). In a nonrandomized, open-label trial spanning 24 months (*n* = 25), 20 patients experienced a 50% reduction in tumor volume and improved pulmonary function after 12 months of sirolimus use (25). Another randomized, double-blind, placebo-controlled, phase 3 trial revealed that sirolimus treatment not only reduced serum Vascular Endothelial Growth Factor-D (VEGF-D) concentration but also enhanced life quality compared to the placebo (26). For extrapulmonary LAM, two reported cases documented a substantial reduction in tumor volume following 12 to months of treatment with another mTOR inhibitor, everolimus, for pelvic LAM (27). These findings underscore the effectiveness of mTOR inhibitors as suppressive treatments for LAM.

It is crucial to acknowledge that Lymphangioleiomyomatosis (LAM) predominantly affects women of childbearing age. Consequently, understanding its implications for pregnancy is of paramount importance to guide reproductive decisions. Pregnant patients with LAM may experience heightened complications, as elevated estrogen levels during pregnancy can exacerbate the condition (28). In light of this, guidelines recommend that patients with LAM carefully consider pregnancy (29). While the teratogenic effects of mTOR inhibitors remain unconfirmed, some animal models suggest associations with intrauterine fetal death and potential excretion in breast milk (7). Contrastingly, a retrospective study indicated that the pregnancy outcomes of women with LAM are comparable to those of normal women. Although pregnancy may impact lung function, it does not necessarily progress to dyspnea or compromise quality of life (30). Successful pregnancies have been reported in LAM patients undergoing long-term mTOR inhibitor treatment, with normal child development despite intermittent exposure to low-dose sirolimus in the early and mid-fetal stages (31). According to the American College of Obstetricians and Gynecologists, undergoing surgery at any stage of pregnancy does not expose the mother or fetus to risks beyond those associated with the disease itself, nor does it pose more complications than in nonpregnant individuals (32). Therefore, we posit that pregnancy may be feasible for stable LAM patients under specific clinical conditions. Nevertheless, the long-term safety implications of surgery and medication on both mothers and fetuses warrant further investigation.

In conclusion, we present a novel case of lymphangioleiomyomatosis with retroperitoneal masses during pregnancy. This condition can easily be mistaken for gynecological tumors, metastatic neoplasms, and other retroperitoneal tumors, thereby posing a substantial challenge for clinicians in diagnosis and differential diagnoses. In the case of pregnant women, it is imperative to comprehensively consider both maternal and fetal safety when formulating treatment strategies. The decision regarding pregnancy should be made based on specific conditions. This case report, detailing a rare disease, contributes significantly to assisting obstetricians and gynecologists in differentiating, diagnosing, and treating pregnancies complicated by LAM.

## Data availability statement

The datasets presented in this article are not readily available because of ethical/privacy restrictions. Requests to access the datasets should be directed to the corresponding authors.

## Ethics statement

Written informed consent was obtained from the participant/patient(s) for the publication of this case report.

## Author contributions

YZ: Writing – original draft. CW: Writing – review & editing. JD: Writing – review & editing. YB: Writing – review & editing. HH: Writing – review & editing. LH: Funding acquisition, Supervision, Writing – review & editing. MY: Writing – review & editing. MM: Writing – review & editing. JC: Data curation, Supervision. YW: Data curation, Methodology, Supervision, Writing – review & editing.
